# Efficacy of potassium polycitrate on renal stone and microlithiasis predisposed by metabolic disorders

**DOI:** 10.22088/cjim.8.4.296

**Published:** 2017

**Authors:** Hadi Sorkhi, Nazanin Saeedizand, Mohammad Poornasrollah, Ali Bijani, Hamid Shafi

**Affiliations:** 1 Non-Communicable Pediatric Diseases Research Center, Health Research Institute, Babol University of Medical Sciences, Babol.; 2 Student Research Committee, Babol University of Medical Sciences, Babol, Iran.; 3 Non-Communicable Pediatric Diseases Research Center, Health Research Institute, Babol University of Medical Sciences, Babol, Iran.; 4 Social Determint Health Research Center, Health Research Institute, Babol University of Medical Sciences, Babol, IR Iran.

**Keywords:** Renal stone, Potassium polycitrate, Hypercalciuria, Hyperuricosuria, Metabolic disorders, Children less than 2 Years.

## Abstract

**Background:**

According to high prevalence of renal stone in children, we evaluated the efficacy of treatment with potassium citrate and its correlation with metabolic disorders in children less than two years of age with renal stone and microlithiasis.

**Methods:**

In this cross- sectionaly study, 100 patients (less than 2 years old) with renal stone or microlitiasis were evaluated for metabolic disorders. They were treated with potassium citrate and followed-up by ultrasonography every 3 months. Then response to treatment was evaluated according to the fresence metabolic disorders (A p-alue<0.05 was significant).

**Results:**

According to this study, hyperuricosuria and hypercalciuria were the two major metabolic disorders (40-33%). Seventy three patients had complete response, and also there as not a significant difference (p<0.05) in the efficacy of treatment due to types of metabolic disorders. There was significant difference between relative response to treatment in children who had metabolic disorders and patient without any metabolic disorders.

**Conclusion:**

Based on our results the use of potassium citrate in all children less than 2 years with renal stone or microlithiasis especially those with metabolic disorders, are recommended.

**N**ephrolithiasis is common yet an important problem in children. Its prevalence has increased because of change of diet, lifestyle and consumption of some drugs. The prevalence of nephrolithiasis is 1.7–4.1% in females and 4-9% in males (1, 2). The prevalence of renal stone has increased five times in recent years (3). About 40% of children with renal stone have a positive familial history (4). Renal stone disease is less common in children than adults, and it is more commonly associated with metabolic disorders or anatomical malformation of the urinary tract (5). Although the underlying cause cannot always be identified, infections, diet and environmental factors may influence stone formation in all age groups (6). One of the most important aspects in pediatric urolithiasis is the different clinical manifestation. Some infants and younger children may be asymptomatic and older children may present with abdominal pain and nonspecific symptoms (7). The purpose of treatment in children is to diminish the risk of stone formation, preserving the kidney function and decreasing the rate of recurrence. Although sometimes surgical intervention may be needed (8). Medical treatments of nephrolithiasis are as follows: increase fluid intake, restric sodium, and use analgesics in cases of which pain persisted (9). 

Potassium citrate is one of the drugs used in nephrolithias is and its efficacy is being investigated. Citrate prevents the formation of calcium deposits. Also, it inhibits calcium oxalate crystal that can be a nucleus for renal calculi. Plus, citrate increases the urine pH and prevents the formation of uric acid and cysteine stones (10). In the study by Zamonarski et al., potassium citrate was completely safe in therapeutic dosage, in addition, they found that the physiological citrate level in urine is more than other inhibitor agents and therapeutic dosage may cause only a mild metabolic alkalosis. Improvement was seen in 70-75% of cases in one year (11). In a cohort study by Tekin et al., it was observed that 1 mEq/kg potassium citrate per day decreased stone recurrence and normalized urinary citrate level in children 1-15 years old (12).

Most studies are about kidney stone prevention in children and adults (13, 14). There are limited investigations about the efficacy of potassium citrate in children with metabolic disorders (15, 16). Regarding the complications of pediatric nephrolithiasis, a non-invasive, inexpensive and accessible treatment like potassium citrate can be useful. In this study, we investigated the effect of potassium polycitrate on and the correlation between metabolic abnormalities renal stone in children less than 2 years old.

## Methods

In this cross- sectional study, all patients less than two years old who were referred to nephrology clinic and department and had renal stone or microlitiasis (deposits < 3 mm) in ultrasonography were enrolled. Twenty five patients with urinary tract infection, cystic fibrosis and anatomical abnormalities were excluded. Complete metabolic evaluation plus blood urine nitrogen (BUN), creatinine, sodium, potassium, vein blood gas, calcium, phosphate, alkaline phosphatase, uric acid were done. The random urine sample was checked for calcium, creatinine, uric acid and citrate level.

Hypercalciuria was defined if early morning calcium/creatinine ratio was >0.8, >0.6 and <0.21 in neonates, 6-12 months and 1-2 years, respectively. Uric acid to creatinine ratio more than 1 was defined hyperuricosuria. Additionally, the oxalate to creatinine ratio <147 and <72 in children less than 6 months while 7-24 months were normal, respectively. Hypocitraturia was defined as citrate to creatinine ratio of less than 0.51. The existence of urine cysteine was evaluated qualitatively by cyanide-nitroprusside test. Medical treatment with potassium poly citrate (citric acid 22% and potassium citrate 6.88%) 1 ml/kg was done. The dosage of drug was decreased or increased according to urine pH (between 6-7). Moreover, in children with hypercalciuria, hydrochlorothiazide (1-2 mg/kg) was prescribed to achieve the normal level of urine calcium. Patients were followed-up by ultrasonography every 3-4 months. (patients ultrasonography was performed by the same radiologist who evaluated the patients for the first time). The information was collected based on the number or size of stone or microlithiasis, age, sex, and the kind of metabolic disorder. Response to medication was assessed as relative or complete response to potassium polycitrate treatment. Relative response included the reduction of number and size of stone to 30% and complete response was defined if all stones disappeared, too. The information was analyzed by Kaplan-Meier method of which the significant level was set at 0.05. 

## Results

In this study, the data from the 100 patients who were less than 2 years old with renal stone or microlithiasis confirmed by ultrasonography were analyzed. Fifty-eight percent were boys and forty-two percent were girls. The mean age of patient was 6.9±4.9 months. The mean size of stones was 2.5±0.8 mm and the mean number of stone was 3.4±2.6. Fifty-two percent of the patients had one side of the body and forty eight percent had both sides of urolithiasis.

In 200 units of kidney, fifty-two (26%) units did not have any stone or microlithiasis and 148 (74%) units had nephrolithiasis. Among the 100 subjects who were studied, the most common metabolic abnormality were hyperuricosuria (40%) followed by hypercalciuria (33%), hyperoxaluria (28%), hypocitraturia (18%) and cystinuria (2%). Twenty-two percent of patients did not have any known metabolic abnormalities and 36% had more than one metabolic disorder. All the patients were treated with potassium poly citrate and were followed-up by ultrasonography, seventy-three percent had complete response and the relative response was seen in 14% of patients. Thirteen percent of children did not have any response to medication. Table 1 shows the frequency of metabolic disorders and the rate of response to potassium polycitrate in both sexes. 

**Table 1:** Frequency of renal stone or microlithiasis due to metabolic disorders and response to potassium polycitrate in children less than 2 years

**Frequency and relative frequency**	**Relative response**	**Complete response**	**Negative response**	**Metabolic disorder**
40 (40) 19 (45.2) 21 (36.2)	7 (17.5) 2 (10.5) 5 (23.8)	30 (75) 16 (84.2) 14 (66.7)	3 (7.5) 1 (5.3) 2 (9.5)	Hyperuricosuria (total) Female Male
33 (33) 20 (47.6) 13 (22)	6 (18.2) 18 (90) 5 (38.5)	26 (78.7) 1 (5) 8 (61.5)	1 (3) 1 (5) 0	Hypercalciuria (total) Female Male
28 (28) 16 (38) 12 (20)	3 (11) 1 (4) 2 (16.7)	25 (89) 15 (96) 10 (83.3)	- 0 0	Hyperoxaluria (total) Female Male
18 (18) 4 (9.5) 14 (24)	5 (27.5) 1 (25) 4 (28.5)	11 (61.3) 2 (50) 9 (64.4)	2 (11) 1 (25) 1 (7.1)	Hypocitraturia (total) Female Male
2 (2) 1 (2.3) 1 (1.7)	0 0 0	1 (50) 1 (100) 0	1 (50) 0 1 (100)	Cystinuria Female Male
22 (22) 5 (12) 17 (29.3)	3 (13.7) 0 3 (17.7)	12 (54.5) 3 (60) 9 (52.9)	7 (31.8) 2 (40) 5 (29.4)	Without known metabolic disorders

In 22% of patients, any known metabolic abnormality was not found and among them, the rate of response to treatment was 73%. Twenty-seven percent had no response to medication, 36% of patients had more than one metabolic disorder. In this study, we investigated the possible differences in metabolic abnormalities between boys and girls. However, we did not find significant differences in prevalence of all the investigated metabolic parameters (table 1).

**Figure 1:**
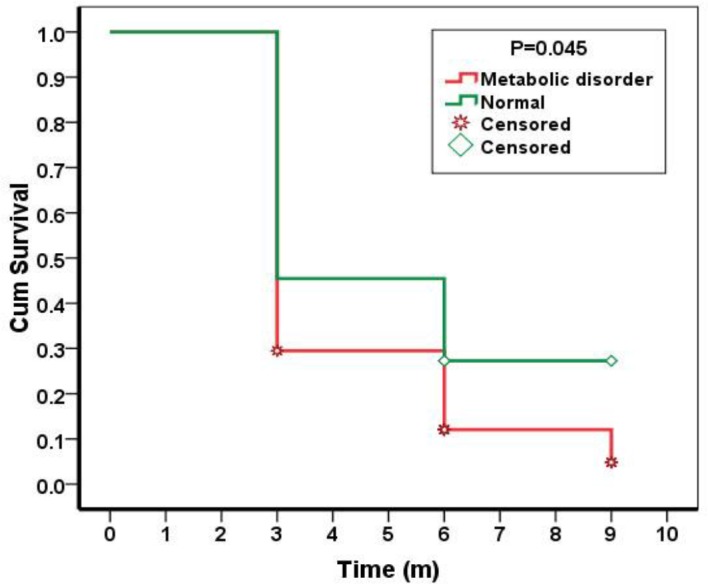
Response to potassium polycitrate according to positive or negative metabolic disorder in children less than 2 years old with renal stone or microlithiasis

According to COX regression results for relative or complete response to treatment, factors such as of age, sex, kind of metabolic disorder, size of stones, renal stone on one side and multiple stones on both sides did not have significant difference (table 2). The only significant difference was more relative response to treatment in children who had metabolic disorders in comparison with patient who did not have any known metabolic abnormalies (P=0.045) (figure 1).

**Table 2:** Multivariant COX regression results for relative and complete response to potassium polycitrate in children less than 2 years old with renal stone or microlithiasis

**P value**	**Complete response** **H.R (Cl 95%)**	**P-value**	**Relative response** **H.R (Cl 95%)**	**Parameter**
0.876	0.95(0.56-1/63)	0.632	0.88 (0.54-1.44)	Sex
0.84	0.77 (0.57-1.03)	0.293	0.87 (0.68-1.12)	Stone Size
0.152	1.44(0.87-2.40)	0.278	1.28 (0.81-2.03)	Hypercalciuria
0.316	1.29 (0.78-2.14)	0.340	1.26 (0.78-2.03)	Hyperoxaluria
0.709	0.88 (0.46-1/69)	0.681	1.12 (0.638-1.99)	Hypocitraturia
0.763	1.07 (0.67-1.71)	0.418	1.19 (0.77-1.82)	Hyperuricosuria
0.104	0.56 (0.28-1.12)	0.442	0.77 (0.41-1.47)	Multistone
0.82	0.93 (0.5-1.73)	0.531	1.19 (0.685-2.08)	Stone in Both sides

## Discussion

According to this study, the most common metabolic abnormality was hyperuricosuria (40%) and then hypercalciuria (33%). Furthermore, about 73% of patients had complete response to polycitrate-K and 13% of patients had not response to drug. In about 20% of children, no metabolic disorders were detected. In one study done on 151 infants (under1year old) in south of Iran, the mean age of patients was 5.46 months and the most metabolic abnormality was hypercalciuria (79%) (17). In Elmaci et al.’s study on 143, 2-6 year-old children, 83.3% of patients had metabolic abnormalities. Hyperuricosuria (24.5%) was the most common abnormality followed by hypercalciuria (21%) (18). Naseri et al. reported 42.7% of metabolic abnormalities in 142 children, 17.6% of them with hypercalciuria and 16.1% of patients had hyperuricosuria. Further, 11.2% of children had multiple metabolic abnormalities (19). Camberari et al. divided all children into two groups, there under and above 10 years old in which metabolic abnormalities were seen in 75% of children under 10 years old and in 60% of children more than 10 years old. Hypercalciuria was the most inherited metabolic disorder in children more than 10 years old and hypocitraturia was the major inherited metabolic disorder in children under 10 years old (20). According to these studies, hypercalciuria and hyperuricosuria were the most common metabolic abnormalities in children with renal stone. The difference of these causes may be due to culture, race, nutrition and geography. Likewise, the difference of age groups in these studies may be another cause of different metabolic abnormalities. Nonetheless, hypercalciuria and hyperuricosuria were the most common abnormalities and further studies may be needed to evaluate them. The male to female ratio of this study was 1.4 .Tekin et al. reported 1.67 and in Sarica’s study, the ratio was 1.36 (12, 21).

The risk of renal stone was higher in males in all studies and more attention and follow-up may be needed in male children. About half of the children had bilateral kidney involvement. In Tekin’s study; about 30% of patients had bilateral involvement (12).Renal stones in both kidneys may be due to metabolic disorders. In this study, 87% of patients had response to polycitrate-K in which 73% of them had complete response and 13% did not have any response. Patients with hyperoxaluria had the best response. In addition, children with metabolic abnormality had better response to drug than the patients without any detected abnormality (91%vs72%).

In Sorkhi et al.’s study done on children with mean age of 36.7±37.4 month, 78.7% of patients had complete response to polycitrate-K (22). Tekin et al. reported decrease of renal stone recurrence in children with hypocitraturia who were treated with polycitrate-K (12). In another study by Naseri et al., complete response was seen in 50% of patients with renal stone and hypercalciuria treated with citrate-K and hydrochlorothiazide (23). Volkan showed significant decrease of renal stone recurrence in 17 children with cystinuria who were treated with high volume fluid intake, restricted of salt and citrate-K (24). Majority of studies were done to prevent renal stone and this study showed the effective treatment of renal stone with polycitrate-K in children less than 2 years of age. 

In conclusion according to this study, hyperuricosuria and hypercalciuria seem to be the most important metabolic factors of nephrolithiasis in children less than 2 years old. Moreover, according to high response to polycitrate-K in this study, we recommend the use of this drug in all children less than 2 years with renal stone or microlitiasis.
